# Comparative risk of delirium among opioid users for non-cancer pain: a retrospective cohort study

**DOI:** 10.1186/s12916-026-04626-0

**Published:** 2026-01-31

**Authors:** Carlos Raul Ramirez Medina, Mark Lunt, William G. Dixon, Meghna Jani

**Affiliations:** 1 Centre for Epidemiology Versus Arthritis, Centre for Musculoskeletal Research, The University of Manchester, Stopford Building, Oxford Road, Manchester, M13 9PT UK; 2 Division of Informatics, Imaging and Data Science, The University of Manchester, Manchester, UK; 3 NIHR Manchester Biomedical Research Centre, Manchester University NHS Foundation Trust, Manchester Academic Health Science Centre, Manchester, UK; 4 Department of Rheumatology, Salford Royal Hospital, Northern Care Alliance, Salford, UK

**Keywords:** Analgesics, Opioid, Opiate, Delirium, Pain management, Early warning score, Electronic health records, MME, Opioid-related harms, Oxycodone

## Abstract

**Background:**

Opioid use for chronic non-cancer pain remains common in the UK, despite limited evidence of long-term effectiveness. Delirium, a serious acute confusional state associated with increased mortality, is a known adverse effect of opioid use. Pharmacological differences between opioids may influence delirium risk, but comparative evidence is scarce. This study evaluated the association of opioid type and dosage with the risk of in-hospital delirium in non-cancer patients.

**Methods:**

We conducted a retrospective cohort study using electronic health records (EHRs) from a tertiary care hospital in northwest England (September 26, 2014–December 31, 2020). Adult (≥ 18 years) without cancer who were administered with opioids during admission were included. Delirium was identified using the 4 ‘A’s Test or through a combination of ICD-10 codes and new-onset confusion scores (= 3) on the National Early Warning Score. Daily opioid doses were converted to daily morphine milligram equivalents (MME/day) to assess the effect of dose across different opioid types. Incidence rates were calculated by opioid type and opioid dosage. Cox regression models, adjusted for confounders, were used to evaluate delirium risk.

**Results:**

Among 50,586 opioid-exposed patients (mean [SD] age, 55 [20] years; 53% female), 867 patients (1.7%) experienced delirium during their first hospital admission (mean [SD] age, 75.1 [16.7] years). Compared to codeine, oxycodone (hazard ratio [HR] 3.52, 95% *C*I 2.77–4.46), fentanyl (*HR* 2.45, 95% *CI* 1.71–3.51), buprenorphine (*HR* 2.43, 95% *CI* 1.54–3.82), combination opioids (*HR* 2.22, 95% *CI* 1.63–3.02), and morphine (*HR* 2.15, 95% *CI* 1.65–2.79) were associated with significantly higher delirium risk. No clear dose–response association was observed: doses of 50–119 MME/day were not associated with a significant increase in risk compared to < 50 MME/day (*HR* 0.96, 95% *CI* 0.66–1.39).

**Conclusions:**

Using in-hospital medication administration records to capture opioid exposure, we found that oxycodone, fentanyl, buprenorphine, morphine, and combination opioids were associated with increased delirium risk compared with codeine. Oxycodone was associated with a higher risk of delirium compared with both codeine and morphine. These findings support personalised opioid prescribing in non-cancer pain and can inform shared clinical decision-making to prevent delirium in patients prescribed opioids.

**Supplementary Information:**

The online version contains supplementary material available at 10.1186/s12916-026-04626-0.

## Background

Opioids remain commonly prescribed for non-cancer pain in the UK [1], particularly for musculoskeletal (MSK) conditions [2], despite concerns about their limited long-term efficacy and associated risks. Among the most serious adverse events is delirium, an acute confusional state linked to increased mortality, long-term cognitive impairment, and higher risks of hospital-acquired complications [3].

Opioid use is a well-established risk factor for delirium [4], with studies showing a twofold increased risk in patients with advanced cancer [5], as well as in medical and surgical populations [4]. Although the overall risk of opioid-associated delirium is recognised [6], it may vary by opioid type due to differences in pharmacokinetic and pharmacodynamic properties [7]. Significant differences in risk between opioid types have been reported for other adverse outcomes, including all-cause mortality, constipation, and opioid abuse [8–10]; however, evidence comparing delirium risk across specific opioid types remains sparse. Delirium is estimated to have cost the NHS in England £10.8 billion in 2022 [11], prolonging hospital stays and increasing mortality [12], yet up to one-third of cases are considered preventable [13].

Despite its clinical importance, delirium has been less extensively studied than other opioid-related harms [14], primarily due to diagnostic challenges and frequent under-recognition [15]. Defined as a serious acute neuropsychiatric syndrome characterised by inattention and cognitive impairment, delirium can be triggered by multiple factors, including acute illness, surgery, trauma, and drug exposure [16].

Previous work identifying delirium as an outcome using hospital data has shown that relying on ICD-10 codes alone has high specificity but poor sensitivity [17]. A Canadian hospital study found that reliance on ICD-10 codes alone yielded high specificity, identifying delirium in only 6.3% of admissions using ICD-10 codes, compared with 25.7% identified through chart review [17]. These findings underscore the value of integrating validated delirium screening tools, such as the 4AT (4 ‘A’s Test: Alertness, Abbreviated Mental Test-4, Attention, and Acute change or fluctuating course) [12], and electronic National Early Warning Scores (NEWS) in addition to ICD-10 codes that offer a valuable opportunity to improve case identification beyond what was previously possible.

Hospital data that include in-hospital medication administration records can further improve the accuracy of exposure assessment and reduce the risk of misclassification bias compared with the more common reliance on prescription or dispensing data, providing more reliable estimates of opioid-adverse outcomes associations. Reducing exposure misclassification is especially important for medications commonly prescribed ‘as required’ for pain management, such as opioids, where a large proportion of prescriptions may have flexibility in the number of tablets administered [18].

To date, no large-scale, high-quality comparative studies have evaluated the risk of delirium across different opioid types [7]. Most existing evidence has been derived from small US cohort studies with limited sample sizes, primarily focused on cancer-related pain, and often missing important details about opioid use, such as route of administration, dose, and timing [7].

The aim of this study was to evaluate the comparative risk of new-onset opioid-induced delirium in hospitalised non-cancer patients administered opioids, by opioid type and dosage, using large-scale electronic health records from a UK tertiary hospital.

## Methods

### Study design and setting

We conducted a retrospective cohort study using secondary care electronic health records (EHRs) from a large tertiary care hospital in the northwest of England between September 26, 2014, and December 31, 2020. Records included opioid-prescribing and administration data, clinical assessments, diagnoses, and discharge summaries. The study period was selected to ensure both the 4AT and the electronic National Early Warning Score (NEWS), a tool for assessing acute patient deterioration, were consistently implemented in the EHR, allowing inclusion only of patients with these measures recorded.

### Study population

We included adults (≥ 18 years) who were hospitalised during the study period, who received opioids, and who had no prior history of cancer. Patients with current or recent malignancy (within 2 years), same-day discharges, or hospital admissions exceeding 91 days were excluded. Same-day discharges were excluded as they may not allow sufficient time to measure inpatient opioid exposure or capture incident delirium. Very long hospital stays and patients with cancer were also excluded, as these represent distinct patient groups with different underlying mechanisms and baseline risks of delirium. Eligible admissions were required to have at least one NEWS assessment recorded. The index date was defined as the first opioid administration, and follow-up was censored at the earliest of: first delirium event, discharge, death, or study end.

### Data preparation for opioid administration (exposure)

Opioid administration data were prepared using inpatient electronic prescribing records, which included the drug name, the administration route, the dosage, and the timing. Exposure was time-varying and categorised as either monotherapy (specifically codeine, tramadol, morphine, fentanyl, buprenorphine, oxycodone, and less commonly prescribed opioids such as diamorphine, dihydrocodeine, methanol, hydromorphone, and pethidine) or opioid combination therapy. Missing data were imputed using related fields. Opioid doses were grouped by the route of administration (e.g. oral, injection, or transdermal) and converted into daily morphine milligram equivalents (MME/day) using CDC conversion factors [19] to assess the effect of dose across different opioid types.

Total opioid exposure was categorised into clinically meaningful thresholds [1] as follows: low (< 50 MME/day), moderate (50–119 MME/day), and high (≥ 120 MME/day), as defined previously [1, 10]. Delirium events were attributed to opioid exposure for up to 1 day after the final recorded date. Crude incidence rates were calculated by opioid use status, opioid type, and MME/day. Details of the full data preparation and MME calculations are provided in the supplementary methods (Additional File).

### Outcome

In-hospital delirium events were defined using either a 4AT score ≥ 4 (indicating delirium) or a recorded ICD-10 code for delirium and a high new-onset confusion score (= 3) on a NEWS assessment. For cases identified using the second definition (ICD-10 + NEWS), the date of the delirium event was defined as the date of the NEWS assessment, as ICD-10 codes are recorded at discharge. If a patient met both criteria, the earlier of the two dates was used to define the onset of delirium. Only the first delirium episode for each admission was included in the analysis.

### 4AT testing

The 4AT has become a standard in clinical practice in the UK for identifying patients with probable delirium in acute hospital settings and emergency departments. Scored from 0 to 12, a 4AT score ≥ 4 indicates possible delirium, with or without cognitive impairment [12].

We analysed 4AT assessments recorded during each eligible admission in the study period. Completion of a 4AT assessment was defined as having at least one recorded 4AT score during a hospitalisation. As 4AT assessments may not be conducted for all patients, we broadened our outcome definition to also include cases identified through a combination of ICD-10 discharge codes for delirium and a high new-onset confusion score (= 3) on the NEWS.

### NEWS assessment

NEWS, developed by the Royal College of Physicians, is the UK’s most widely used early warning system for detecting clinical deterioration in adults [20]. Endorsed by NHS England for use in acute and ambulance settings, guidelines recommend monitoring all adult inpatients with NEWS at least every 4 h after admission [21].

### Covariates

Baseline characteristics, including age, sex, body mass index (BMI), ethnicity, and the Index of Multiple Deprivation (IMD), were recorded at hospital admission. Comorbidities, including chronic kidney disease (CKD), MSK disorders (such as inflammatory and non-inflammatory joint diseases, spinal conditions, soft tissue disorders, connective tissue diseases, and bone disorders), multiple sclerosis, Parkinson’s disease, dementia, liver disease, and a history of alcohol excess, were identified using ICD-10 diagnostic codes (Additional File: Supplementary Table 1) in hospital discharge records within the 2 years preceding and including the admission of interest. Potential confounders, including age, CKD, a history of alcohol excess, liver disease, and dementia, were adjusted for, as they were associated with both the opioid type being administered (exposure) and delirium (outcome) but were not on the causal pathway (Additional File: Supplementary Fig. 1). Effect modifiers included BMI < 20, major or orthopaedic surgery, and the presence of a serious infection on admission. Serious infections were identified using ICD-10 discharge codes during the admission of interest, while major or orthopaedic surgeries were defined based on OPCS discharge codes.

### Statistical analysis

In this analysis, only the first admission episode per patient was included. Crude incidence rates for delirium were calculated by opioid type and MME/day. Cox regression models were used to evaluate the association between opioid exposure and delirium risk. The primary ‘opioid-type model’ compared delirium risk across opioid types, using codeine as the referent (with a separate comparison using morphine as the referent). Secondary analyses included an ‘opioid dose model’, which examined the relationship between time-varying daily MME levels and delirium incidence. Both unadjusted and adjusted models were fitted (as described in the section above). Stratified analyses were performed to assess the impact of potential effect modifiers, including major/orthopaedic surgery, infection, and low body mass index (BMI < 20) at admission. A complete case analysis was used for the BMI-stratified analysis, as BMI data were available for only 53% of the cohort.

Sensitivity analyses included the following: (1) A complete case analysis using delirium defined solely by 4AT scores (≥ 4), (2) an alternative outcome definition based on a high new-onset confusion score (= 3) in the NEWS assessment combined with delirium ICD-10 codes, and (3) an additional model adjusting for BMI where data were available. All statistical analyses were conducted in R (version 4.1.3).

### Ethics

The study received ethics approval from the Health Research Authority (Reference 21/EM/0147, East Midlands Leicester South).

## Results

### Study population and baseline characteristics

**Fig. 1:**
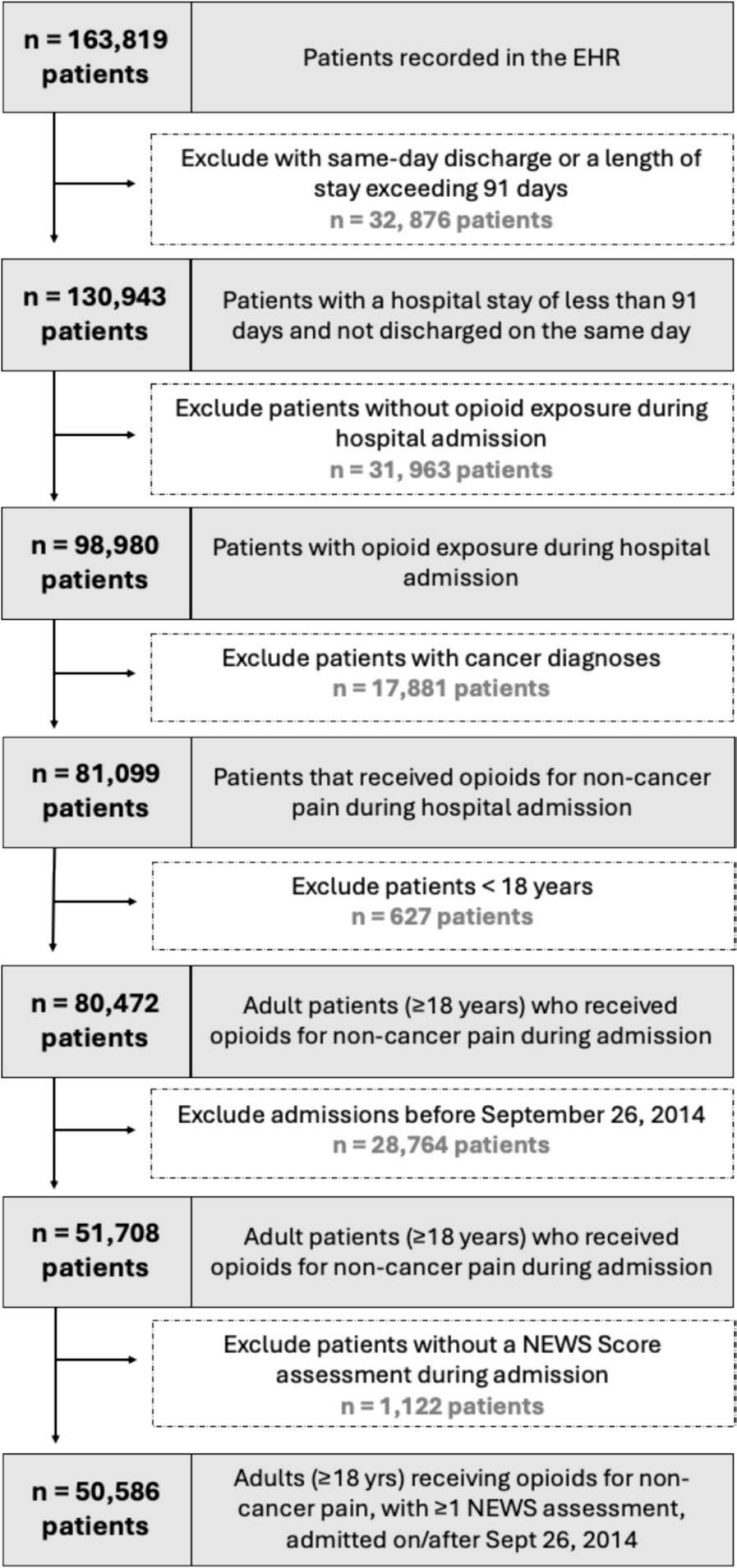
Cohort derivation diagram

Within the study window, 50,586 patients treated with opioids for non-cancer pain met the inclusion criteria and were included in the analysis (mean [SD] age, 55 [20] years) (Fig. 1). Baseline characteristics are summarised in Table 1. Just over half of the cohort were female (*n* = 26,878; 53%), and the majority were identified as being of White Caucasian ethnicity (*n* = 46,763; 92%). Nearly half (*n* = 22,053; 44%) had a musculoskeletal condition, and 13,550 (27%) patients were in the most deprived socioeconomic category at the time of admission.

**Table 1:** Baseline characteristics by opioid drug type at initiation

Characteristic	Total	Codeine	Tramadol	Morphine	Fentanyl	Buprenorphine	Oxycodone	Other	Combination
	*N* = 50,586	*N* = 17,241	*N*= 1346	*N* = 15,128	*N* = 2027	*N* = 423	*N* = 6636	*N* = 107	*N* = 7678
Age, mean [SD] years	55 (20) years	53 (20) years	60 (17) years	51 (19) years	65 (18) years	73 (17) years	71 (17) years	61 (15) years	50 (19) years
Age group									
18–29	6704 (13%)	2696 (16%)	83 (6.2%)	2275 (15%)	107 (5.3%)	63 (15%)*	172 (2.6%)	23 (22%)*	1361 (18%)
30–49	14,170 (28%)	4970 (29%)	278 (21%)	5215 (34%)	310 (15%)		676 (10%)		2645 (34%)
50–64	12,005 (24%)	4112 (24%)	415 (31%)	4175 (28%)	432 (21%)	48 (11%)	997 (15%)	37 (35%)	1789 (23%)
65–79	10,892 (22%)	3562 (21%)	401 (30%)	2254 (15%)	761 (38%)	113 (27%)	2450 (37%)	33 (31%)	1318 (17%)
> 80	6815 (13%)	1901 (11%)	169 (13%)	1209 (8.0%)	417 (21%)	199 (47%)	2341 (35%)	14 (13%)	565 (7.4%)
Sex									
Female	26,878 (53%)	8887 (52%)	801 (60%)	7642 (51%)	1124 (55%)	286 (68%)	3938 (59%)	60 (56%)	4140 (54%)
Male	23,703 (47%)	8352 (48%)	545 (40%)	7485 (49%)	903 (45%)	137 (32%)	2697 (41%)	47 (44%)	3537 (46%)
Ethnicity									
White	46,763 (92%)	15,638 (91%)	1273 (95%)	13,984 (92%)	1917 (95%)	408 (96%)	6306 (95%)	103 (96%)	7134 (93%)
Asian	1941 (3.8%)	795 (4.6%)	45 (3.3%)	570 (3.8%)	68 (3.4%)	NA	203 (3.1%)	NA	249 (3.2%)
Black	924 (1.8%)	406 (2.4%)	19 (1.4%)	271 (1.8%)	21 (1.0%)	NA	60 (0.9%)	NA	143 (1.9%)
Mixed	320 (0.6%)	150 (0.9%)	NA	94 (0.6%)	18 (0.8%)	NA	14 (0.2%)	NA	50 (0.7%)
Other ethnic group	523 (1.0%)	207 (1.2%)	NA	172 (1.1%)		NA	41 (0.6%)	NA	86 (1.1%)
Indices of multiple deprivations									
1 (Most deprived)	13,550 (27%)	4895 (28%)	405 (30%)	4143 (27%)	420 (21%)	99 (23%)	1473 (22%)	38 (36%)	2077 (27%)
2	7739 (15%)	2628 (15%)	209 (16%)	2359 (16%)	272 (13%)	66 (16%)	990 (15%)	19 (18%)	1196 (16%)
3	5697 (11%)	1919 (11%)	147 (11%)	1719 (11%)	242 (12%)	58 (14%)	752 (11%)	12 (11%)	848 (11%)
4	5114 (10%)	1795 (10%)	145 (11%)	1521 (10%)	170 (8.4%)	37 (8.7%)	651 (9.8%)	13 (12%)	782 (10%)
5	3745 (7.4%)	1175 (6.8%)	102 (7.6%)	1076 (7.1%)	198 (9.8%)	42 (9.9%)	583 (8.8%)	NA	562 (7.3%)
6	3223 (6.4%)	1063 (6.2%)	89 (6.6%)	968 (6.4%)	142 (7.0%)	29 (6.9%)	429 (6.5%)	NA	500 (6.5%)
7	2947 (5.8%)	931 (5.4%)	75 (5.6%)	876 (5.8%)	151 (7.4%)	17 (4.0%)	453 (6.8%)	NA	441 (5.7%)
8	3337 (6.6%)	1054 (6.1%)	88 (6.5%)	957 (6.3%)	162 (8.0%)	46 (11%)	529 (8.0%)	NA	499 (6.5%)
9	2642 (5.2%)	873 (5.1%)	50 (3.7%)	769 (5.1%)	138 (6.8%)	25 (5.7%)*	410 (6.2%)	NA	379 (4.9%)
10	2119 (4.2%)	739 (4.3%)	28 (2.1%)	589 (3.9%)	109 (5.4%)		332 (5.0%)	NA	312 (4.1%)
Comorbidities									
Diabetes (type 1 or 2)	6557 (13%)	2116 (12%)	258 (19%)	1567 (10%)	365 (18%)	97 (23%)	1369 (21%)	21 (20%)	764 (10.0%)
Chronic kidney disease	3331 (6.6%)	855 (5.0%)	98 (7.3%)	515 (3.4%)	262 (13%)	70 (17%)	1241 (19%)	12 (11%)	278 (3.6%)
Musculoskeletal (MSK) conditions	22,053 (44%)	6705 (39%)	825 (61%)	6038 (40%)	1146 (57%)	250 (59%)	3759 (57%)	56 (52%)	3274 (43%)
Multiple sclerosis	276 (0.5%)	108 (0.6%)	22 (1.6%)	70 (0.5%)	NA	NA	32 (0.5%)	NA	28 (0.4%)
Parkinson’s disease	442 (0.9%)	133 (0.8%)	12 (0.9%)	95 (0.6%)	30 (1.5%)	12 (2.8%)	119 (1.8%)	41 (0.5%)*	
Liver disease (mild and severe)	728 (1.4%)	252 (1.5%)	27 (2.0%)	238 (1.6%)	29 (1.4%)	NA	85 (1.3%)	89 (1.1%)*	
Alcohol excess	5856 (12%)	2258 (13%)	118 (8.8%)	1866 (12%)	164 (8.1%)	39 (9.2%)	506 (7.6%)	905 (12%)*	
Dementia	1867 (3.7%)	427 (2.5%)	37 (2.7%)	425 (2.8%)	155 (7.6%)	78 (18%)	627 (9.4%)	118 (1.5%)*	
Major or orthopaedic surgery during admission	8443 (17%)	2120 (12%)	103 (7.7%)	2993 (20%)	550 (27%)	NA	957 (14%)	NA	1711 (22%)
Infection during admission	6638 (13%)	2769 (16%)	211 (16%)	1840 (12%)	189 (9.3%)	86 (20%)	620 (9.3%)	22 (21%)	901 (12%)
BMI < 20 during admission	3106 (6.1%)	885 (5.1%)	54 (4.0%)	892 (5.9%)	157 (7.7%)	52 (12%)	714 (11%)	11 (10%)	341 (4.4%)
Hospitalisation length, mean [SD] days	9 (13) days	8 (12) days	7 (11) days	8 (12) days	12 (15) days	14 (17) days	14 (16) days	10 (13) days	7 (10) days
Most common route	Oral	Oral	Oral	Oral	Parenteral	Topical (patch)	Oral	Oral	Oral
Morphine milligram equivalent (MME)/day at initiation									
[0–50)	43,807 (87%)	~ 17,240 (99%)*	~ 1340 (99%)*	11,804 (78%)	761 (38%)	384 (91%)	6348 (96%)	~ 100 (98%)*	5820 (76%)
[50–120)	352 (7%)	NA*	NA*	1531 (10%)	352 (17%)	NA*	226 (3%)	NA*	1233 (16%)
120 +	3427 (7%)	NA*	NA*	1793 (12%)	914 (45%)	32 (8%)	62 (1%)	NA*	625 (8%)

*NA* not available

*Records have been grouped or replaced with ‘NA’ for statistical disclosure control to protect patient confidentiality

A history of alcohol excess was recorded in 5856 patients (12%), and dementia was recorded in 1867 patients (3.7%). The mean hospital stay for the overall cohort was 9 days (*SD* = 13). Patients initiated on codeine and morphine were younger than those initiated on other opioids (Table 1).

Opioid dosing at initiation varied, with most patients (*n* = 43,807, 87%) receiving a lower dose (< 50 MME/day). Patients in this group were generally older (mean [SD] age 56 [20] years) than those in the 50–119 MME/day (47 [17] years) and the ≥ 120 MME/day (51 [17] years) groups (Additional File: Supplementary Table 2). Additionally, 15% of patients in the < 50 MME/day group were aged ≥ 80, compared to only 2.9% and 3.5% in the 50–119 and the ≥ 120 MME/day groups, respectively. The < 50 MME/day group also had higher proportions of patients with dementia (4.2% vs 0.3% and 0.4%), CKD (7.2% vs 2.6% and 3.1%), and infection as the primary admission diagnosis (14% vs 9.6% and 4.9%). In contrast, major or orthopaedic surgery was more common among those initiated on higher doses: 37% in the ≥ 120 MME/day group and 23% in the 50–119 MME/day group, compared to 15% in the < 50 MME/day group (Additional File: Supplementary Table 2).

The most prescribed opioid varied by initial dose. Among patients receiving lower doses (< 50 MME/day) at initiation (*n* = 43,807), codeine was the most frequently prescribed opioid (*n* = 17,240; 40%). Conversely, at moderate (50 to < 120 MME/day) and high (≥ 120 MME/day) doses, morphine was the most prescribed (45% and 52%, respectively).

Overall, 737 opioid users (3.4%) developed delirium (mean [SD] age, 74.8 [15.13] years) throughout the study period. Of these, 867 patients (1.7%) experienced delirium during their first hospital admission, which formed the basis for the analyses in this study. Among those who experienced delirium, the mean length of hospital stay was 23 days (*SD* = 20).

### Completion rates of 4AT and NEWS score assessments

A 4AT assessment was completed at least once for 39,408 unique patients (78%) during the study period, with 1089 unique patients (2.1%) having a recorded score > 4, indicative of delirium. Additionally, ICD-10 primary and secondary diagnostic discharge codes were used alongside NEWS scores to identify potential delirium events. NEWS assessments were available for patients from September 26, 2014, onwards, and were recorded for 98% of the hospitalisations throughout the study period.

Both a NEWS score and a delirium ICD-10 code during admission were identified for 1061 unique patients (2%) during the study period. Using the composite definition as described in the outcome section, 1737 unique patients (3.4%) were identified as patients who experienced delirium during the hospital admission throughout the study period.

### Incidence rates

The overall incidence rate of delirium while on an opioid was 3.27 per 1000 person-days. Oxycodone had the highest incidence rate (6.03 per 1000 person-days) (Table 2). Fentanyl and buprenorphine also showed relatively high incidence rates (4.8 per 1000 person-days and 3.7 per 1000 person-days, respectively) (Table 2). Tramadol had the lowest incidence rate (1.24 per 1000 person-days) among opioid users in our cohort.

**Table 2:**
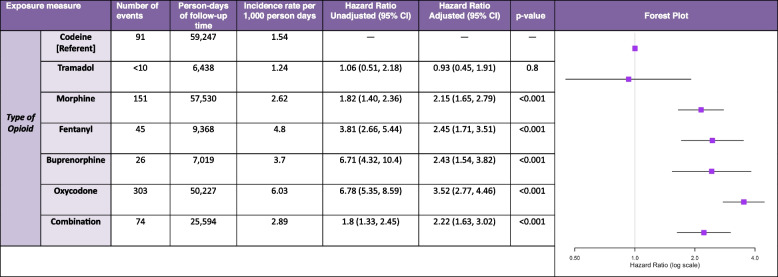
Association between administered opioid exposure and delirium

^*^Other opioids (diamorphine, dihydrocodeine, methanol, hydromorphone, pethidine) were excluded from this table due to a low count

### Comparative risk of delirium associated with different opioids

Compared with codeine, after adjustment for possible confounders, oxycodone (hazard ratio [HR] 3.52, 95% *CI* 2.77–4.46), fentanyl (*HR* 2.45, 95*% CI* 1.71–3.51), buprenorphine (*HR* 2.43, 95% *CI* 1.54–3.82), and morphine (*HR* 2.15, 95% *CI* 1.65–2.79) were significantly associated with a higher risk of delirium in the adjusted opioid drug type analysis. Tramadol (*HR* 0.93, 95% *CI* 0.45–1.91) was not associated with a significantly different risk when compared with codeine. Full results for the association between the comparative risk of delirium with various opioid types are presented in Table 2.

Compared to morphine, patients administered oxycodone showed a significantly higher risk of experiencing delirium in the adjusted opioid drug type analysis (*HR* 1.64, 95% *CI* 1.33–2.02, *P* < 0 0.001; Additional File: Supplementary Table 3). There was a significantly lower risk of delirium associated with tramadol and codeine compared to morphine (*HR* 0.43, 95% *CI* 0.21–0.88, *P* < 0.05 and *HR* 0.47, 95% *CI* 0.36–0.61, *P* < 0.001, respectively; Additional File: Supplementary Table 3).

### MME/day dosage

There was low variability in MME/day dosage across the study period, with most patients (87%) consistently receiving a low dose (< 50 MME/day) (Table 1).

In the adjusted MME analysis, patients receiving < 50 MME/day had a crude incidence rate of 3.61 events per 1000 person-days compared to 1.46 events per 1000 person-days among those receiving 50–119 MME/day. A daily morphine milligram equivalent (MME) dose of 50–119 did not show a significant increase in risk compared to lower doses [0–50) (*HR* 0.96, 95% *CI* 0.66–1.39; Table 3).

**Table 3:**
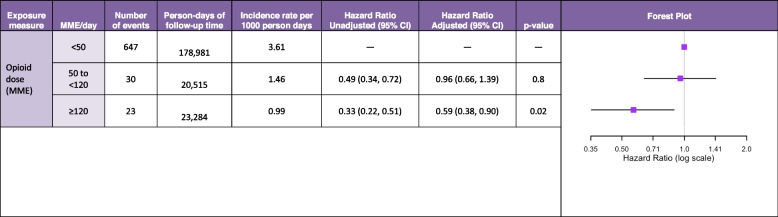
Association between morphine milligram equivalents per day thresholds and delirium

### Sensitivity analyses

Sensitivity analyses examining opioid type showed consistent results, with oxycodone, fentanyl, morphine, and combination opioids associated with a significantly higher risk of delirium compared to codeine (Additional File: Supplementary Results). In the first analysis, limited to complete cases with available 4AT scores, oxycodone had the strongest association (*HR* 2.95, 95% *CI* 2.15–4.04, *p*-value < 0.001). In the second analysis, which included a definition of ICD-10 codes and NEWS only, oxycodone was associated with the highest risk of delirium (*HR* 4.54, 95% *CI* 3.21–6.40, *p*-value < 0.001). In the third adjusted analysis restricted to patients with available BMI data, oxycodone again showed the highest hazard ratio (*HR* 2.23, 95% 1.65–3.03, *p*-value < 0.001) (Additional File: Supplementary Table 4).

For opioid dosage (MME/day), the findings were consistent across all sensitivity analyses: no significant association was observed between higher doses of opioids (≥ 50 MME/day) and increased delirium risk when using < 50 MME/day as the reference (Additional File: Supplementary Table 5).

In the stratified analyses, oxycodone showed a consistent higher risk in non-infection, infection, and low-BMI patients; fentanyl, morphine, buprenorphine, and opioid combinations were also associated with increased risk in several stratified analyses (Additional File: Supplementary Table 6).

## Discussion

In this study of hospitalised patients administered opioids for non-cancer pain, oxycodone, fentanyl, buprenorphine, morphine, and combination opioids were associated with a significantly higher risk of delirium compared to codeine. Tramadol did not confer an increased risk after adjusting for confounding factors. When compared to morphine, oxycodone was associated with a significantly higher risk, whereas tramadol and codeine were associated with a significantly lower risk. In the categorical analysis of MME/day using clinically meaningful thresholds, a dose of 50–119 MME/day did not show a significant increase in risk compared to doses under 50 MME/day.

These findings derived from a large population support the notion that differences in pharmacological profiles of opioids may lead to differential risks, especially between oxycodone and an increased risk of delirium in patients administering opioids for non-cancer pain.

Drug-induced delirium is thought to result from dopaminergic overactivity and cholinergic underactivity [16]. Oxycodone acts primarily on µ-opioid receptors within dopaminergic pathways, particularly the nigrostriatal and mesolimbic dopaminergic systems [22], and may pose a higher risk of dopaminergic dysregulation. This is supported by evidence that different opioid types carry differential risks to the dopamine system [23]. These findings are especially important as patients undergoing major or orthopaedic surgery are commonly prescribed oxycodone in both younger and older patients [24]. In contrast, buprenorphine has been reported to have a lower impact on dopamine D2-like receptor sensitivity [25] and may thus present a reduced potential for dopaminergic-related adverse effects compared to opioids such as oxycodone or morphine. One study in 114 patients initiating injectable morphine, oxycodone, or fentanyl found a significantly lower incidence of delirium in those receiving fentanyl, compared to the oxycodone and morphine groups, aligning with our findings [26]. However, large-scale evidence linking oxycodone to an increased delirium risk remains limited, with few directly comparable studies available. Existing research is often of low quality [7], with small sample sizes or a focus on different populations, such as individuals receiving palliative care or those with cancer-related pain. A 2017 systematic review found no compelling evidence to support a differential risk of delirium based on opioid type in elderly patients, citing methodological issues such as low-quality studies with a high risk of selection bias, small sample sizes, and inconsistent delirium definitions [7]. A study in a small cohort of 259 patients with cancer receiving opioids compared the incidence of delirium between opioid types and found no significant difference in delirium incidence between oral morphine, oxycodone, and tapentadol [27]. However, due to the small differences in incidence rates and the limited sample size, the study may not have had sufficient power to detect a statistically significant difference between the opioid types.

Tramadol was not associated with a significantly increased risk compared with codeine. A Spanish study that included 368,960 participants found no significant difference in delirium risk with tramadol, consistent with our findings, despite associations with higher all-cause mortality, cardiovascular events, and fractures [28]. It is important to note, however, that in our study, few patients received this medication (2.7%) as inpatients, so this could reflect a lack of statistical power.

A previous systematic review assessing the comparative risk of delirium with different opioid types found six studies of poor (*n* = 5) or moderate (*n* = 1) quality in postsurgical contexts, highlighting that information on dose, route, and timing of administration was often missing [7]. In our study, we were able to address these concerns with accurate information on administered dose and duration. However, unlike other opioid-associated adverse events that appear to be dose dependent [9, 10], we did not find a strong association between daily opioid dose and the incidence of delirium. The findings, namely a nonsignificant decrease in risk at 50–119 MME/day and a significant decrease in risk for doses > 120 MME/day compared to < 50 MME/day, together with wide confidence intervals, are likely due to relatively few patients escalating to higher doses, resulting in fewer observed delirium events. For the outcome of delirium, differences between opioid types appear to be more important than dose. There are a few possible explanations for the lack of a clear dose effect. First, most patients in our cohort maintained a stable low-dose regimen, possibly reflecting cautious opioid-prescribing practices or limited need for dose escalation in non-cancer pain. Second, patients receiving lower opioid doses (< 50 MME/day) tended to be older, had a higher prevalence of comorbidities (such as dementia and CKD), and presented with higher rates of infections at admission, all well-recognised risk factors for delirium (Additional File: Supplementary Table 1). These baseline characteristics may have contributed to clinicians’ reluctance to escalate opioid doses in this higher-risk group. By contrast, patients receiving higher opioid doses (> 50 MME/day) were more likely to have undergone major or orthopaedic surgery during admission and were generally younger and less comorbid (Additional File: Supplementary Table 1). These differences in baseline risk and clinical context likely meant that patients were channelled to lower daily doses, resulting in a lower observed risk of delirium in the highest-dose group. This pattern more likely reflects the avoidance of higher doses in patients perceived to be at greater risk of delirium, rather than a genuinely lower risk associated with high-dose opioid therapy. A third explanation is that opioid type may have a greater impact on delirium risk than dose. Due to pharmacological differences between opioids, patients susceptible to delirium may develop it relatively soon after a potential putative opioid drug was commenced, even at low doses. The patient would develop delirium, leading to cessation of the drug and without the opportunity for further dose escalation.

As highlighted by a previous systematic review [7], previous studies have not evaluated the effect of time-varying daily doses on delirium. It is not common for studies on opioid-related adverse events to report MME/day [7], as it is challenging to prepare such data with a combination of ‘as-required’ dosing, missing prescribing or dispensing data, and overlapping medications [18].

### Strengths

To our knowledge, this is the first large-scale UK study to evaluate the comparative risk of opioid-induced delirium in non-cancer pain using administered drug data while also assessing the impact of daily dose. A key strength of this study is the use of medication administration records rather than prescribing data. Prescriptions do not guarantee that a patient received the drug, as prescriptions may not be dispensed or taken by the patient. Administration data provide a more accurate measure of actual exposure, reduce the risk of exposure misclassification bias compared with prescribing data [18], and provide more reliable estimates of drug-outcome associations.

In addition, delirium was identified using scores from a validated tool (4AT), completed during admission by clinical staff, ensuring a standardised and more reliable outcome definition. We also assessed the effect of (BMI < 20) in those patients with available BMI records at admission, as low BMI may be a contributor to frailty.

### Limitations

These findings should be interpreted in light of certain limitations, many of which are inherent to retrospective observational research, including unmeasured confounding. Established risk factors of delirium, such as frailty scores, poor sleep, malnutrition, pain severity [29], and prior episodes of delirium, were not consistently recorded in the EHR during the study period. The study also did not account for the influence of time-varying concomitant medications such as antidepressants and benzodiazepines, which may also affect delirium risk [30]. Pre-hospital opioid exposure was not available, preventing assessment of whether patients had prior opioid use. BMI data records were frequently incomplete, as BMI may not always be routinely measured in all admissions. Therefore, a complete case analysis was performed for the pre-mentioned stratified analyses, reducing the sample size.

Although our case definition combined ICD-10 discharge codes, 4AT screening, and NEWS scores to improve sensitivity and specificity compared with ICD-10 codes alone, the case definition could not be formally validated. The ethics approvals in place designed to protect patient anonymity and reduce the risk of re-identification prevented access to the raw data or individual patient records by researchers.

Another important limitation was the small number of patients in the higher-dose categories, as well as limited representation of certain opioid drug types, reflecting prescribing practices in the UK. Previous work has shown important opioid-prescribing differences between the UK and other jurisdictions in North America [31]. For instance, hydrocodone and hydromorphone are less commonly prescribed in the UK, with higher doses at initiation prescribed in North America [31]. As mentioned earlier, the apparent lower risk at higher doses is unlikely to represent a true biological effect and may instead reflect a combination of cautious prescribing practices and channelling bias.

Finally, we excluded patients who were admitted for less than 24 h, as, on further evaluation, they were found to be day-case dialysis patients or those undergoing minor procedures (e.g. joint injections). Also, such patients had incomplete data on medication administration depending on how many hours they were in hospital. Similarly, patients who had admissions longer than 91 days (3 months) were excluded, as very long admissions may include patients with complex medical needs awaiting social care placements or patients who are in hospital for prolonged recovery post intensive care and are not reflective of the majority of patients being admitted to hospital. Such patients, despite being few in our cohort, may have an even higher baseline risk of delirium.

### Wider implications

This study represents a step forward in understanding the differential risks of opioids causing delirium among patients with non-cancer pain, with important implications for both clinical practice and public health in the UK. Given the limited pain-relief options available, these results support a more personalised approach to pain management to reduce the risk of delirium. Our results suggest that oxycodone carries the highest risk across all analyses. Improving reporting and diagnosis of opioid-related adverse effects such as delirium, increasing awareness, and proactively managing delirium as a potential consequence of opioid therapy can improve patient outcomes and reduce complications during hospital care.

### Conclusions

A difference in delirium risk between opioid types was observed, with oxycodone associated with a higher risk compared with codeine and morphine in patients with non-cancer pain. Using hospital EHRs and administered drug data, our study — the largest to date on this research area — aimed to minimise exposure misclassification and to identify delirium events using the standardised, validated 4AT assessment tool. These findings support personalised opioid prescribing in non-cancer pain, aiming to inform shared decision-making to prevent delirium in hospitalised patients with the risk factors highlighted in this study.

## Supplementary Information

Additional file 1: Supplementary Methods: Data preparation steps for processing and converting electronic administration data into daily opioid doses. Supplementary Table 1: ICD-10 codes for covariates used in the study. Supplementary Fig. 1: Directed Acyclic Graph demonstrating potential effect of confounders and effect modifiers. Supplementary Table 2: Baseline characteristics by opioid dosage at initiation. Supplementary Table 3. Main analysis—Comparative Risk of Delirium Associated with Different Opioids (morphine as reference). Supplementary Table 4. Association between administered opioid exposure & delirium (sensitivity analyses). Supplementary Table 5. Association between Morphine Milligram Equivalents per day thresholds & delirium (stratified analyses). Supplementary Results: Stratified analyses, including patients undergoing major or orthopaedic surgery, with and without serious infection during admission and BMI < 20. Supplementary Table 6. Association between administered opioid exposure & delirium (stratified analyses).

## Data Availability

The data that support the findings of this study are available from the Northern Care Alliance, but restrictions apply to the availability of these data, which were used under license for the current study, and so are not publicly available. Data are however available from the authors upon reasonable request and with permission of the Northern Care Alliance [https://www.northerncarealliance.nhs.uk/contact-us]. The data for the study were accessed and analysed within a Secure Research Environment that is hosted by Northern Care Alliance. In order to access the data, researchers will need to be Office of National Statistics Safe Researcher accredited [https://www.ons.gov.uk/aboutus/whatwedo/statistics/requestingstatistics/secureresearchservice/becomeanaccreditedresearcher] and need to have undergone appropriate background checks prior to permission being granted from the host organisation.

## Abbreviations

4A: 4 ‘A’s Test: Alertness, AMT4 (Abbreviated Mental Test-4), Attention, and Acute change/fluctuations
BMI: Body mass index
CI: Confidence interval
CKD: Chronic kidney disease
HR: Hazard ratio
IMD: Index of Multiple Deprivation
EHRs: Electronic health records
MME: Morphine milligram equivalents
MSK: Musculoskeletal
NEWS: National Early Warning Scores
NA: Not available
SD: Standard deviation
