# Supplementary material for: Comparative risk of delirium among opioid users for non-cancer pain: a retrospective cohort study
Source: BMC Med. 2026 Jan 31;24:90. doi: 10.1186/s12916-026-04626-0 (PMC12892522; doi:10.1186/s12916-026-04626-0)
Supplement: Supplementary file 1 — Additional file 1: Supplementary Methods: Data preparation steps for processing and converting electronic administration data into daily opioid doses. Supplementary Table 1: ICD-10 codes for covariates used in the study. Supplementary Fig. 1: Directed Acyclic Graph demonstrating potential effect of confounders and effect modifiers. Supplementary Table 2: Baseline characteristics by opioid dosage at initiation. Supplementary Table 3. Main analysis—Comparative Risk of Delirium Associated with Different Opioids (morphine as reference). Supplementary Table 4. Association between administered opioid exposure & delirium (sensitivity analyses). Supplementary Table 5. Association between Morphine Milligram Equivalents per day thresholds & delirium (stratified analyses). Supplementary Results: Stratified analyses, including patients undergoing major or orthopaedic surgery, with and without serious infection during admission and BMI < 20. Supplementary Table 6. Association between administered opioid exposure & delirium (stratified analyses). [file 12916_2026_4626_MOESM1_ESM.docx]

# Supplementary Material

**Additional File: Supplementary Methods**

Data preparation steps for processing and converting electronic administration data into daily opioid dose

The dataset used for this study provided detailed information about administered drugs, including opioids. The dataset included unique patient identifiers, medication names (sometimes including dosage and strength), dates of prescription and administration, dosage instructions, and medication routes. Columns also capture the administered dose, strength, and frequency of administration, among other details.

1. ***Data Cleaning***
   1. Filter data within study window
   2. Remove non-administered medications (if performed date was missing or task status code indicated it was not performed).
   3. Extract medication class and details (including drug strength, administration route, form, and unit of measurement).
   4. Exclude medications that are not opioids (based on a predefined list of opioid names).
   5. Ensure consistency in unit of measurement.
   6. Clean administration routes
   7. Clean strength data
   8. Clean dose - In cases like co-codamol (which contains both codeine and paracetamol), remove the paracetamol component and ensure the dose is accurately recorded based on the codeine portion.
2. ***Data Imputation***
   1. Impute missing dose (if dose is missing, but low and high dosage are available, compute the mean of these two values to impute the missing dose).
   2. Impute missing unit of measures based on other available data (e.g., check medication name for possible information).
   3. Impute Missing Strength Information: use the data from Medication Name or unit of measure to infer the correct strength.
   4. Impute missing route: use the available medication name or route code to impute the route of administration.
3. **Data Transformation**
   1. Split Data by Route of Administration: Separate the data based on the route of administration (e.g., Oral, Injection, Transdermal patches) so that calculations for each can be handled appropriately.
   2. Process Oral Medications: For each unique combination of patient id, date, and medication class, compute the daily dose and MME
   3. Process Injection/Continuous Infusion
   4. Process Transdermal Patches.
4. ***MME Conversion Factors***
   1. Calculate MME/day by multiplying the daily dose of each opioid by the corresponding analgesic conversion factor, as specified by the CDC, considering the frequency, duration, and route-specific considerations. One MME is equivalent to one milligram of morphine.
5. The final dataset contained cleaned and transformed data, including the following:
   1. Unique identifier for each patient.
   2. Date of medication administration.
   3. Name of the opioid administered.
   4. The total dose administered on that day.
   5. The total daily MME corresponding to the administered dose.

**Additional File, Supplementary Table 1: ICD-10 codes for covariates used in the study.**

| **Covariate** | **ICD-10 Codes Included** |
| --- | --- |
| **Alcohol excess** | E512, F10, F100–F109, G312, K70, K701–K704, K709, K852, K860, R780, T51, T510, T519, X45, X450–X453, X455–X459, X65, X650–X653, X655–X659, Y15, Y150–Y153, Y155–Y159, Y911–Y913, Y919, Z714, Z721 |
| **Liver disease** | B18, B19, I850, I859, I864, I982, K700–K704, K709, K711, K713–K715, K717, K720, K721, K722, K729, K730–K739, K740–K749, K750, K751, K754, K760, K762–K764, K765–K767, K768, K769, Z944 |
| **Dementia** | A810, F00–F03, F051, F1027, G30, G310–G311, G318 |
| **Cancer** | C00–C09, C10–C26, C30–C41, C43–C58, C60–C96, C7A, C7B, D00–D09, D10–D49, D3A |
| **Diabetes** | E08–E13 |
| **Musculoskeletal** | M00–M99 |
| **Chronic Kidney Disease (CKD)** | N18 |

**Additional File Supplementary Figure 1: Directed Acyclic Graph demonstrating potential effect of confounders and effect modifiers**


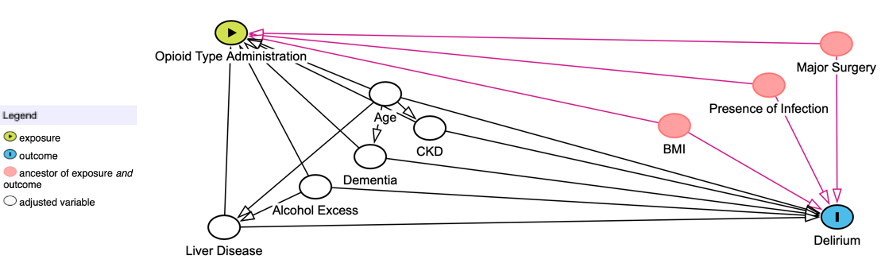


Potential confounders—including age, chronic kidney disease (CKD), liver disease, history of alcohol excess, and dementia—were adjusted for in the analysis, as they were associated with both opioid type (exposure) and delirium (outcome), but not on the causal pathway. Effect modifiers included major or orthopaedic surgery, BMI <20, and the presence of a serious infection on admission.

**Additional File Supplementary Table 2. Baseline characteristics by opioid dosage at initiation**

| **Characteristic** | **[0 - 50)** | **[50 - 120)** | **120+** |
| --- | --- | --- | --- |
| Age, Mean (SD) | 56 (20) | 47 (17) | 51 (17) |
| Female | 23,474 (54%) | 1,701 (51%) | 1,703 (50%) |
| Male | 20,329 (46%) | 1,651 (49%) | 1,723 (50%) |
| Age group |  |  |  |
| 18-29 | 5,742 (13%) | 547 (16%) | 414 (12%) |
| 30-49 | 11,669 (27%) | 1,365 (41%) | 1,134 (33%) |
| 50-64 | 10,101 (23%) | 831 (25%) | 1,072 (31%) |
| 65-79 | 9,693 (22%) | 512 (15%) | 686 (20%) |
| >80 | 6,598 (15%) | 97 (2.9%) | 120 (3.5%) |
| Diabetes | 5,851 (13%) | 328 (9.8%) | 378 (11%) |
| CKD | 3,137 (7.2%) | 87 (2.6%) | 107 (3.1%) |
| MSK | 18,799 (43%) | 1,482 (44%) | 1,771 (52%) |
| IBS | 757 (1.7%) | 78 (2.3%) | 54 (1.6%) |
| Hypothyroidism | 2,324 (5.3%) | 119 (3.6%) | 125 (3.6%) |
| Sclerosis | 249 (0.6%) | 13 (0.4%) | 14 (0.4%) |
| Parkinson | 415 (0.9%) | 10 (0.3%) | 17 (0.5%) |
| Crohn | 545 (1.2%) | 78 (2.3%) | 98 (2.9%) |
| Muscular Dystrophy | 39 (<0.1%) | 2 (<0.1%) | 6 (0.2%) |
| Dementia | 1,846 (4.2%) | <10 (0.3%) | 12 (0.4%) |
| Infection as primary diagnosis | 6,147 (14%) | 322 (9.6%) | 169 (4.9%) |
| Diverticulitis | 662 (1.5%) | 33 (1.0%) | 37 (1.1%) |
| Frailty | 709 (1.6%) | <10 (0.3%) | 21 (0.6%) |
| BMI < 20 | 2,801 (6.4%) | 147 (4.4%) | 157 (4.6%) |
| Major/Orthopaedic Surgery | 6,414 (15%) | 777 (23%) | 1,252 (37%) |
| Hospitalisation Length,  Mean (SD) | 9 (13) | 8 (11) | 9 (11) |

## **Additional File Supplementary Table 3. Main analysis - Comparative Risk of Delirium Associated with Different Opioids (morphine as reference)**

| **Exposure measure** | | **Hazard Ratio Adjusted (95% CI)** | **p-value** |
| --- | --- | --- | --- |
| Type of opioid | Morphine [Referent] | **—** | **—** |
|  | Tramadol | 0.43 (0.21, 0.88) | 0.021 |
|  | Codeine | 0.47 (0.36, 0.61) | <0.001 |
|  | Fentanyl | 1.14 (0.81, 1.60) | 0.4 |
|  | Buprenorphine | 1.13 (0.73, 1.76) | 0.6 |
|  | Oxycodone | 1.64 (1.33, 2.02) | <0.001 |
|  | Combination | 1.03 (0.73,1.76) | 0.6 |

**Additional File Supplementary Table 4. Association between administered opioid exposure & delirium (sensitivity analyses)**

| **Sensitivity Analysis** | **Exposure measure** | | **Hazard Ratio Adjusted (95% CI)** | **p-value** |
| --- | --- | --- | --- | --- |
| **4AT Score definition only (Complete Case Analysis)** | Type of opioid | Codeine [Referent] | **—** | **—** |
|  |  | Tramadol | 0.79 (0.28, 2.17) | 0.6 |
|  |  | Morphine | 2.37 (1.67, 3.37) | <0.001 |
|  |  | Fentanyl | 2.90 (2.12, 3.98) | <0.001 |
|  |  | Buprenorphine | 1.73 (0.88, 3.39) | 0.11 |
|  |  | Oxycodone | 2.95 (2.15-4.04) | <0.001 |
|  |  | Combination | 2.13 (1.41, 3.22) | <0.001 |
| **ICD-10 and NEWS score** | Type of opioid | Codeine [Referent] | — | — |
|  |  | Tramadol | 0.91 (0.33, 2.53) | 0.9 |
|  |  | Morphine | 2.13 (1.47, 3.09) | <0.001 |
|  |  | Fentanyl | 1.81 (1.00, 3.26) | 0.05 |
|  |  | Buprenorphine | 4.50 (2.53, 7.97) | <0.001 |
|  |  | Oxycodone | 4.54 (3.21, 6.40) | <0.001 |
|  |  | Combination | 2.05 (1.32, 3.19) | <0.001 |
| **Analysis with those with BMI data available** | Type of opioid | Codeine [Referent] | — | — |
|  |  | Tramadol | 0.92 (0.37, 2.30) | 0.9 |
|  |  | Morphine | 1.51 (1.08, 2.11) | 0.015 |
|  |  | Fentanyl | 1.85 (1.17, 2.92) | 0.008 |
|  |  | Buprenorphine | 1.84 (1.01, 3.37) | 0.047 |
|  |  | Oxycodone | 2.23 (1.65, 3.03) | <0.001 |
|  |  | Combination | 1.74 (1.19, 2.56) | 0.005 |

**Additional File Supplementary Table 5. Association between Morphine Milligram Equivalents per day thresholds & delirium (stratified analyses)**

| **Stratified Analysis** | **MME/day** | **Person-days of follow-up time** | **Incidence rate per 1000 person days** | **Hazard Ratio Unadjusted (95% CI)** | **Hazard Ratio Adjusted (95% CI)** | **p-value** |
| --- | --- | --- | --- | --- | --- | --- |
| **Major or orthopaedic surgery** | <50 | 24,041 | 2.41 | — | — | — |
|  | 50 to <120 | 4,760 | 1.47 | 0.76 (0.35, 1.66) | 1.67 (0.75, 3.70) | 0.2 |
|  | ≥120 | 6,123 | 0.82 | 0.32 (0.13, 0.81) | 0.63 (0.25, 1.57) | 0.3 |
|  | Not Exposed | 6,562 | 2.59 | 3.56 (1.80, 7.03) | 3.43 (1.77, 6.63) | <0.001 |
|  | | | | | | |
| **Infection** | <50 | 22,978 | 4.87 | — | — | — |
|  | 50 to <120 | 2,132 | 2.35 | 0.63 (0.26, 1.51) | 1.67 (0.75, 3.70) | >0.9 |
|  | ≥120 | 1,998 | 1.50 | 0.48 (0.15, 1.56) | 0.63 (0.25, 1.57) | 0.6 |
|  | Not Exposed | 12,409 | 2.90 | 1.54 (0.94, 2.54) | 3.43 (1.77, 6.63) | 0.4 |
|  | | | | | | |
| **Non-infection** | <50 | 153,689 | 3.46 | — |  | — |
|  | 50 to <120 | 18,167 | 1.38 | 0.48 (0.32, 0.72) | 0.96 (0.64, 1.44) | 0.60 |
|  | ≥120 | 20,978 | 0.95 | 0.33 (0.21, 0.51) | 0.6 (0.38, 0.93) | 0.01 |
|  | Not Exposed | 79,565 | 1.68 | 1.56 (1.22, 2.00) | 1.39 (1.09, 1.77) | 0.02 |
|  | | | | | | |
| **BMI <20** | <50 | 21,649 | 6.14 | — | — | — |
|  | 50 to <120 | 2,463 | 2.44 | 0.55 (0.23, 1.32) | 1.05 (0.43, 2.58) | >0.9 |
|  | ≥120 | 2,647 | 1.51 | 0.34 (0.12, 0.92) | 0.68 (0.25, 1.88) | 0.40 |
|  | Not Exposed | 15,869 | 2.84 | 1.49 (0.99, 2.24) | 1.44 (0.95, 2.19) | 0.12 |

**Additional File Supplementary Results**

*Stratified Analyses*

Compared to codeine, oxycodone was consistently associated with the highest risk of delirium across multiple subgroups. In patients with low BMI (<20), oxycodone showed a hazard ratio of 3.8 (95% CI 2.05–7.03). Among those without serious infection, the hazard ratio was 3.96 (95% CI 3.03–5.19) and in patients undergoing major or orthopaedic surgery, the association was even stronger (HR 6.56, 95% CI 2.53 -17.0; compared to codeine). In contrast, among patients admitted with a primary diagnosis of serious infection, fentanyl was associated with the highest risk (HR 3.45, 95% CI 1.73–6.89), exceeding that of oxycodone. Tramadol consistently showed no significant association with increased delirium risk across all subgroups. Full details are provided below.

*Stratified Analyses*

**Major or Orthopaedic Surgery**

In patients who underwent major or orthopaedic surgery (8,443 patients, 17%), oxycodone was associated with the highest incidence rate of delirium (5.42 per 1,000 person-days). Compared to codeine, only oxycodone (HR 6.56, 95% CI 2.53 -17.0) had a significantly higher risk for delirium in the adjusted opioid drug type analysis (Supplementary Table 6).

**Patients without serious infection during admission**

In patients without infection, oxycodone had the highest incidence rate (6.16 per 1,000 person-days). Oxycodone (HR 3.96, 95% CI 3.03–5.19), buprenorphine (HR 2.68, 95% CI 1.61–4.46), morphine (HR 2.24, 95% CI 1.66– 3.03), and fentanyl (HR 2.21, 95% CI 1.44–3.41), were significantly associated with a higher risk of delirium compared to codeine. Tramadol was not significantly associated with increased risk (HR 0.79, 95% CI 0.32–1.96, P-value 0.6).

**Patients with serious infection during admission**

Among patients with a primary diagnosis for infection (6,638 patients, 13%), fentanyl had the highest delirium incidence rate (14.48 per 1,000 person-days), followed by oxycodone and buprenorphine. Compared to codeine, fentanyl (HR 3.45, 95% CI 1.73–6.89), oxycodone (HR 2.08, 95% CI 1.16–3.73), morphine (HR 1.93, 95% CI 1.12–3.31) and opioid combinations (HR 2.40, 95% CI 1.23–4.71) were significantly associated with a higher risk of delirium. Tramadol (HR 1.20, 95% CI 0.35–4.12, P>0.05) and buprenorphine (HR 1.67, 95% CI 0.62–4.50, P>0.05) did not show a significant association.

**BMI <20**

Among patients with a BMI <20 (3,106 patients, 6.1%), oxycodone had the highest incidence rate (9.87 per 1,000 person-days). Compared to codeine, oxycodone (HR 3.8, 95% CI 2.05–7.03), buprenorphine (HR 3.57, 95% CI 1.47–8.63), morphine (HR 2.67, 95% CI 1.34–5.31), opioid combinations (HR 2.58, 95% CI 1.16–5.74), and fentanyl (HR 2.28, 95% CI 0.90–5.75) were significantly associated with a higher risk of delirium. Tramadol (HR 2.36, 95% CI 0.51–11.0, P>0.05) did not show a significant association (Supplementary Table 6).

**Additional File Supplementary Table 6. Association between administered opioid exposure & delirium (stratified analyses)**

| **Stratified Analysis** | **Exposure measure** | | **Person-days of follow-up time** | **Incidence rate per 1000 person days** | **Hazard Ratio Unadjusted (95% CI)** | **Hazard Ratio Adjusted (95% CI)** | **p-value** |
| --- | --- | --- | --- | --- | --- | --- | --- |
| **Major or orthopaedic surgery** | Type of opioid | Codeine [Referent] | 6,081 | 0.82 | **—** | **—** | **—** |
|  |  | Tramadol | 450 | 0.00 | 0 (0.00, 0.00) | 0 (0.00, 0.00) | **—** |
|  |  | Morphine | 12,454 | 0.96 | 1.44 (0.51, 4.10) | 2.20 (0.77, 6.25) | 0.14 |
|  |  | Fentanyl | 1,562 | 1.28 | 1.42 (0.27, 7.34) | 1.26 (0.25, 6.33) | 0.80 |
|  |  | Buprenorphine | 463 | 0.00 | 0 (0.00, 0.00) | 0 (0.00, 0.00) | **—** |
|  |  | Oxycodone | 8,308 | 5.42 | 13.9 (5.50, 35.3) | 6.56 (2.53, 17.0) | <0.001 |
|  |  | Combination | 5,200 | 1.15 | 1.35 (0.41, 4.42) | 1.51 (0.46, 4.98) | 0.50 |
|  | | | | | | | |
| **Non-infection** | Type of opioid | Codeine [Referent] | 50,153 | 1.38 | — | — | — |
|  |  | Tramadol | 5,258 | 0.95 | 0.88 (0.36, 2.18) | 0.79 (0.32, 1.96) | 0.60 |
|  |  | Morphine | 49,688 | 2.31 | 1.76 (1.30, 2.37) | 2.24 (1.66, 3.03) | <0.001 |
|  |  | Fentanyl | 8,247 | 3.64 | 3.11 (2.02, 4.78) | 2.21 (1.44, 3.41) | <0.001 |
|  |  | Buprenorphine | 5,998 | 3.50 | 7.51 (4.58, 12.3) | 2.68 (1.61, 4.46) | <0.001 |
|  |  | Oxycodone | 44,982 | 6.16 | 7.63 (5.85, 9.95) | 3.96 (3.03, 5.19) | <0.001 |
|  |  | Combination | 22,453 | 2.67 | 1.84 (1.31, 2.60) | 2.26 (1.60, 3.20) | <0.001 |
|  | | | | | | | |
| **Infection** | Type of opioid | Codeine [Referent] | 8,354 | 2.63 | — | — | — |
|  |  | Tramadol | 1,105 | 2.71 | 1.56 (0.46, 5.37) | 1.20 (0.35, 4.12) | 0.80 |
|  |  | Morphine | 6,938 | 4.76 | 2.11 (1.23, 3.63) | 1.93 (1.12, 3.31) | 0.01 |
|  |  | Fentanyl | 1,036 | 14.48 | 8.39 (4.37, 16.1) | 3.45 (1.73, 6.89) | <0.001 |
|  |  | Buprenorphine | 979 | 5.11 | 4.06 (1.51, 10.9) | 1.67 (0.62, 4.50) | 0.30 |
|  |  | Oxycodone | 4,637 | 5.61 | 3.93 (2.20, 7.02) | 2.08 (1.16, 3.73) | 0.02 |
|  |  | Combination | 2,834 | 4.94 | 1.88 (0.96, 3.65) | 2.40 (1.23, 4.71) | 0.01 |
|  | | | | | | | |
| **BMI <20** | Type of opioid | Codeine [Referent] | 6,088 | 1.97 | — | — | — |
|  |  | Tramadol | 574 | 3.48 | 2.34 (0.51, 10.8) | 2.36 (0.51, 11.0) | 0.3 |
|  |  | Morphine | 5,747 | 4.87 | 2.32 (1.18, 4.58) | 2.67 (1.34, 5.31) | 0.004 |
|  |  | Fentanyl | 1,473 | 5.43 | 3.33 (1.35, 8.21) | 2.28 (0.90, 5.75) | 0.031 |
|  |  | Buprenorphine | 1,909 | 5.24 | 7.13 (3.04, 16.7) | 3.57 (1.47, 8.63) | 0.001 |
|  |  | Oxycodone | 7,197 | 9.87 | 6.31 (3.42, 11.6) | 3.8 (2.05, 7.03) | <0.001 |
|  |  | Combination | 2,875 | 4.17 | 2.44 (1.10, 5.44) | 2.58 (1.16, 5.74) | 0.015 |
